# Cognitive decision-making in police use of force: a scoping review based on eye-tracking research

**DOI:** 10.3389/fpsyg.2026.1837475

**Published:** 2026-08-12

**Authors:** Yaoguang Li, Wenlai Cui

**Affiliations:** 1 Graduate School, People’s Public Security University of China, Beijing, China; 2 School of Police Law Enforcement Abilities Training, People’s Public Security University of China, Beijing, China

**Keywords:** cognitive decision-making, eye-tracking technology, police use of force, scoping review, use-of-force decision-making

## Abstract

**Purpose:**

This scoping review aimed to systematically map and synthesize the existing eye-tracking literature on cognitive decision-making in police use of force. Specifically, it integrated evidence across five key domains: eye-tracking metric frameworks, decision-making paradigms, expert–novice differences, stress-related influences, and training interventions. The findings contribute to a more comprehensive understanding of the cognitive mechanisms underlying police use-of-force decision-making and provide empirical support for the optimization of police training systems.

**Methods:**

Following the PRISMA-ScR guidelines, a systematic search was conducted across four databases, including PubMed. Of 103 identified records, 15 studies were included after duplicate removal, title and abstract screening, and full-text assessment. Data on publication characteristics, study designs, participant characteristics, eye-tracking metrics, research themes, and key findings were extracted and descriptively synthesized to map the current evidence landscape and characterize the existing body of research.

**Results:**

The included studies revealed that eye-tracking metrics in police use-of-force decision-making have evolved from single fixation-based measures to multidimensional frameworks incorporating fixation allocation, saccadic dynamics, visual states, and physiological–eye movement coupling. Existing decision-making paradigms highlight the importance of accurate attention allocation to threat-relevant cues and the avoidance of expectation-driven “shooting before seeing” responses. Expert–novice studies suggest that tactical training experience, rather than occupational tenure alone, contributes to more efficient gaze strategies under stress. Stress may impair decision-making by increasing threat expectancy and reducing fixation stability, whereas training interventions targeting deliberate practice and tactical gaze control improve gaze efficiency and response speed, with limited effects on decision accuracy among experienced officers.

**Conclusion:**

Eye-tracking research on police use-of-force decision-making has progressed from descriptive observation to mechanism-based explanation and intervention validation. Future research should integrate cognitive reappraisal and attentional regulation into a unified training framework, translating laboratory findings into scalable police training practices to reduce decision-making errors through evidence-based approaches.

## 1 Introduction

In police practice, use-of-force decision-making is directly related to officer safety, the legal rights of individuals involved, and broader public safety. It plays an irreplaceable role in advancing the standardization of law enforcement, safeguarding judicial fairness, and maintaining public trust (Cunningham et al., 2022; Petersen et al., 2025). However, injuries resulting from police use of force have increasingly emerged as a significant public safety concern. Recent estimates suggest that more than 70,000 individuals are injured annually in such incidents (Petersen et al., 2025). Research by Stroshine et al. (2024) further demonstrates that the costs of unjustified use of force are substantial, as any application of force carries the risk of injury or even death. Moreover, variations in use-of-force decisions across different situational contexts directly shape the trajectory of police–citizen encounters and the severity of resulting harm (Nieuwenhuys et al., 2012). These realities underscore the necessity for law enforcement officers to accurately assess use-of-force scenarios and respond appropriately. Accordingly, gaining a deeper understanding of the cognitive mechanisms underlying police use-of-force decision-making, identifying key factors that contribute to decision biases, and optimizing training interventions are of both theoretical and practical significance.

Unlike conventional decision-making behaviors, police use-of-force decisions represent a complex cognitive process characterized by information acquisition, evaluation, and response selection under uncertainty, severe time constraints, and potential threat. Therefore, the concept of cognitive decision-making is adopted in this review to capture the underlying mechanisms through which officers allocate visual attention, identify threat-relevant cues, construct situation awareness, assess risks, and select appropriate responses. This perspective extends beyond outcome-based approaches that focus solely on behavioral decisions (e.g., shooting versus withholding fire) by elucidating how expertise, stress exposure, and training influence the cognitive processes involved in use-of-force decision-making.

Traditional approaches to evaluating police use-of-force training, assessing decision quality, and managing risk have largely relied on instructor judgment, *post hoc* case reviews, and written examinations. These methods are inherently subjective and fail to capture officers’ dynamic cognitive processes in high-pressure, complex, and rapidly evolving operational environments. As a result, they are limited in their ability to identify critical issues such as omissions in information search, misinterpretation of risk signals, and breakdowns in decision-making logic (Alexander et al., 2024; Stroshine et al., 2024; Flick and Schweitzer, 2025). This “black box” of cognitive decision-making hinders efforts to trace the root causes of risky behaviors, accurately diagnose deficiencies in officers’ use-of-force skills, and identify bottlenecks in decision efficiency. Consequently, it constrains the optimization of police training systems and the precise enhancement of risk management capabilities in use-of-force contexts (Geisler and Allwood, 2018; Callahan and Hayes, 2024).

Eye-tracking technology is a real-time and objective method for monitoring ocular movement behavior. Through the integrated use of near-infrared emission and detection, high-speed video capture, and computer-based image processing and data analysis, it enables precise tracking and recording of eye movement trajectories. Key metrics such as fixation location, fixation duration, saccade amplitude, and scan paths can be quantitatively analyzed, providing direct and measurable evidence for understanding how visual information is selected, processed, and integrated during police use-of-force decision-making (Skaramagkas et al., 2023). To date, eye-tracking technology has been widely and effectively applied in several high-risk domains, including aviation safety, military tactical training, medical practice, and education (Ashraf et al., 2018; Martinez-Marquez et al., 2021). In policing contexts, emerging studies have begun to apply this technology to practical scenarios such as face recognition, in-vehicle systems, and cognitive decision-making in law enforcement (Millen and Hancock, 2019; Huhta et al., 2022; Zahabi et al., 2022; Sudkamp et al., 2025), demonstrating its considerable potential and applicability in the field.

Although previous studies have systematically examined the psychological mechanisms, stress-related factors, and decision biases underlying police use-of-force behavior, existing reviews have primarily focused on behavioral outcomes and their associated determinants. A systematic synthesis of the application of eye-tracking technology to police use-of-force decision-making from a visual cognitive processing perspective remains lacking. Moreover, substantial heterogeneity exists across empirical studies in terms of eye-tracking devices, measurement metrics, experimental paradigms, participant characteristics, and research contexts, limiting the comparability and integration of findings. Furthermore, several critical cognitive mechanisms underlying police use-of-force decision-making remain insufficiently understood, including how visual attention influences shoot/no-shoot judgments, how expertise shapes efficient visual search strategies, how stress alters attentional control processes, and whether training interventions can effectively improve decision-making performance.

Therefore, this scoping review aimed to systematically map the empirical literature on cognitive decision-making in police use of force from an eye-tracking perspective and synthesize current evidence across five key domains: eye-tracking metric frameworks, decision-making paradigms, expert–novice differences, stress-related influences, and training interventions. Specifically, this review addressed the following research questions: (1) What eye-tracking metrics have been employed in existing studies, and what aspects of visual cognitive functioning do these metrics represent? (2) Within shoot/no-shoot decision-making paradigms, how are visual attention characteristics associated with decision biases? (3) What consistent differences exist between expert and novice police officers in visual search strategies and cognitive processing patterns? (4) How does stress influence attentional control and use-of-force decision-making? (5) What effects do different training interventions have on eye-movement behaviors and decision-making performance?

By systematically charting and synthesizing empirical evidence, this review seeks to establish a “visual attention–cognitive processing–behavioral selection” framework for police use-of-force decision-making. The findings provide an evidence-based foundation for understanding the cognitive mechanisms underlying police decision-making, optimizing police training systems, and reducing risks associated with use-of-force decision errors.

## 2 Methods

This scoping review was conducted and reported in accordance with the Preferred Reporting Items for Systematic Reviews and Meta-Analyses Extension for Scoping Reviews (PRISMA-ScR) guidelines (Tricco et al., 2018).

### 2.1 Search strategy

Electronic searches were conducted in PubMed, EBSCO, Web of Science, and Scopus, covering the period from database inception to January 2, 2026. The search focused on the application of eye-tracking technology in police use-of-force decision-making. The search strategy is presented in Table 1, with detailed search strings provided in Appendix 1. In addition, reference lists of relevant reviews and retrieved articles were manually screened to identify supplementary studies. When necessary, study authors were contacted to obtain additional information.

**Table 1:** Search strategy (using PubMed as an example).

Steps	Search query
#1	Search: (((((eye tracking[Title/Abstract]) OR (eye movement[Title/Abstract])) OR (gaze tracking[Title/Abstract])) OR (gaze control[Title/Abstract])) OR (quiet eye[Title/Abstract])) OR (gaze analysis[Title/Abstract])
#2	Search: ((((police[Title/Abstract]) OR (law enforcement[Title/Abstract])) OR (police officer[Title/Abstract])) OR (authorized firearms officer[Title/Abstract])) OR (active-duty police[Title/Abstract])
#3	Search: (((((firearms use[Title/Abstract]) OR (weapon use[Title/Abstract])) OR (use of force[Title/Abstract])) OR (weapon deployment[Title/Abstract])) OR (shoot/do not shoot scenario[Title/Abstract])) OR (firearms operation[Title/Abstract])
#4	#1 AND #2 AND #3

### 2.2 Criteria for selection of studies

The inclusion criteria for this study were as follows: (1) experimental studies examining police use-of-force decision-making or related perceptual–cognitive processes in law enforcement contexts; (2) studies employing laboratory-based or simulated tasks to investigate police decision-making behavior; (3) studies that constructed police decision-making scenarios using virtual reality (VR) or immersive simulation environments and incorporated eye-tracking measures; and (4) empirical studies were included if they were conducted in police decision-making simulations, police training environments, or related experimental settings, and employed eye-tracking technology to assess visual attention, gaze behavior, or cognitive processing mechanisms associated with use-of-force decision-making. Eligible participants included active-duty police officers, police academy cadets, law enforcement trainees, and other experimental samples involved in cognitive decision-making paradigms relevant to police use of force. For studies involving non-police participants, only those employing highly realistic police use-of-force decision-making tasks and examining visual attention and cognitive processing mechanisms relevant to police decision-making were included. Such participants were not considered direct representations of police populations but were included to provide insights into the underlying cognitive mechanisms involved in use-of-force decisions.

The exclusion criteria were as follows: (1) studies that did not employ eye-tracking technology or failed to report objective eye-tracking data; (2) theoretical, conceptual, opinion-based, or review articles lacking original empirical data; and (3) studies involving non-law enforcement populations in contexts not directly related to police use-of-force decision-making.

### 2.3 Data synthesis and analyses

The title and abstract screening and subsequent full-text assessment were independently conducted by two reviewers (YGL and WLC). Any discrepancies between reviewers were resolved through discussion until consensus was reached. Due to the exploratory nature of this scoping review, inter-rater agreement statistics (e.g., Cohen’s kappa coefficient or percentage agreement) were not calculated or reported for the study selection process. No restrictions were applied regarding participants’ age, gender, publication year, or publication language.

Following study selection, a standardized Excel form was developed to extract relevant data. The extracted information included: (1) publication characteristics (title, authors, year of publication, and country); (2) methodological characteristics (study type, research objectives, number of groups, study design, and intervention measures); (3) participant characteristics (sample size, age, gender distribution, and years of service); and (4) outcome characteristics (eye-tracking metrics, their functional roles, and key findings).

### 2.4 Quality assessment

The methodological quality of the included studies was appraised using the Mixed Methods Appraisal Tool (MMAT 2018). The MMAT is applicable for evaluating studies with different research designs, including randomized controlled trials, non-randomized studies, and quantitative descriptive studies. Two reviewers independently conducted the quality appraisal, and any discrepancies in the assessment of individual criteria were resolved through discussion and consensus.

## 3 Results

### 3.1 Study selection

**Figure 1:**
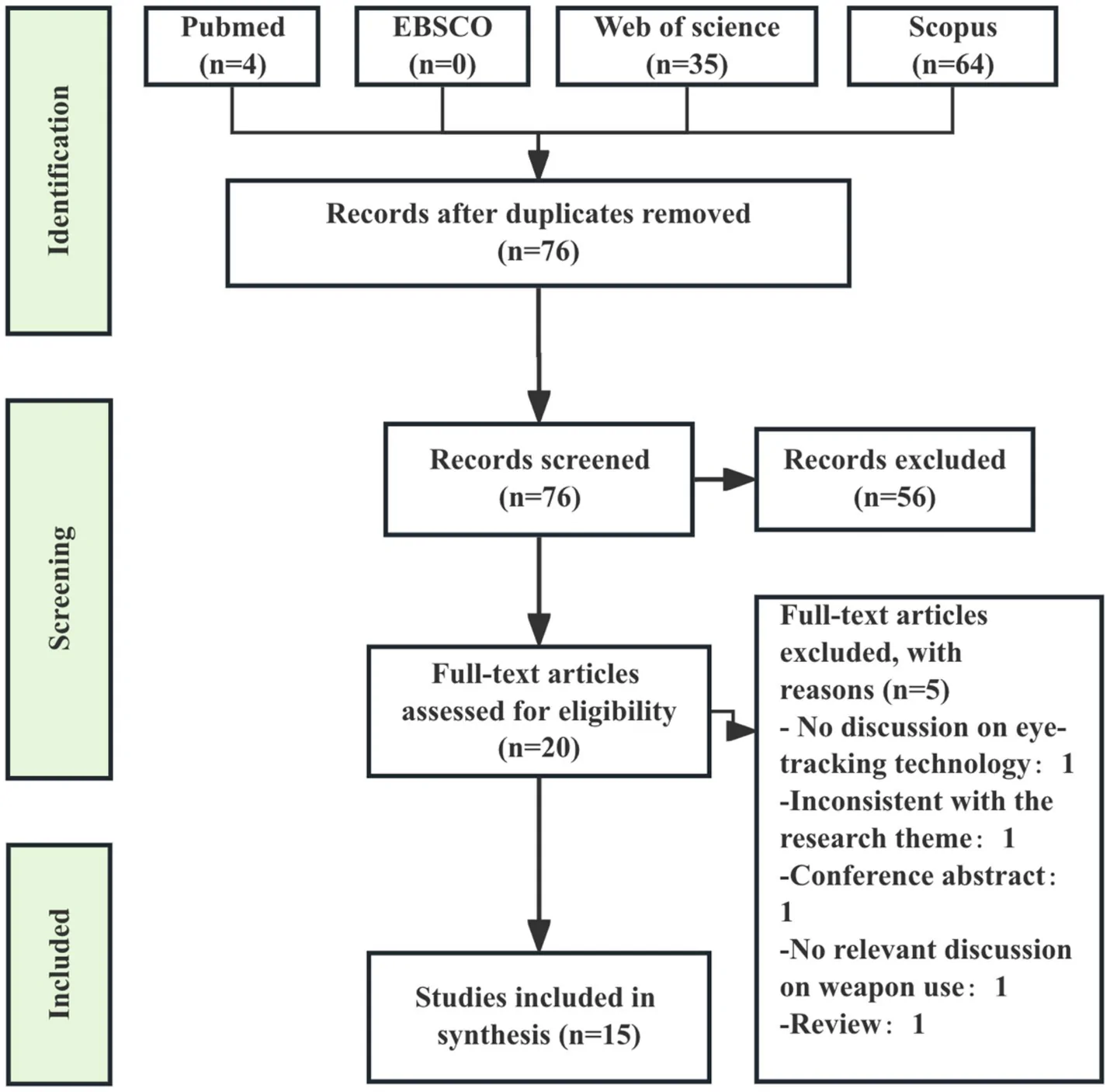
PRISMA flow diagram.

A total of 103 records were identified across the four databases (PubMed: 4; EBSCO: 0; Web of Science: 35; Scopus: 64). After removing duplicates, 76 records remained. Following title and abstract screening, 20 articles were retained for full-text review. Ultimately, 15 studies met the inclusion criteria and were included in the final analysis (Nieuwenhuys and Oudejans, 2010; Nieuwenhuys et al., 2012, 2015; Vickers and Lewinski, 2012; Landman et al., 2016; Martaindale, 2021; Heusler and Sutter, 2022a, 2022b; Huhta et al., 2022; Tawa, 2023; Murray et al., 2024; Olma et al., 2024a, 2024b; Seitchik and Girgenti, 2025; Sudkamp et al., 2025) (see Figure 1).

### 3.2 Characteristics of included studies

#### 3.2.1 Temporal distribution of studies

The 15 studies included in this review were published between 2010 and 2025, spanning a 15-year period and collectively reflecting the developmental trajectory of eye-tracking research on police use-of-force decision-making. In terms of temporal distribution, the period from 2010 to 2016 represents an early exploratory phase (*n* = 5), during which research primarily focused on the effects of stress on shooting performance and initial comparisons between expert and novice officers (Nieuwenhuys and Oudejans, 2010; Nieuwenhuys et al., 2012, 2015; Vickers and Lewinski, 2012; Landman et al., 2016). The years 2020 to 2023 mark a phase of rapid development (*n* = 5), characterized by an expansion of research topics to include training interventions and mechanisms of visual attention (Martaindale, 2021; Heusler and Sutter, 2022a, 2022b; Huhta et al., 2022; Tawa, 2023). Finally, the period from 2024 to 2025 represents a phase of deepening integration (*n* = 5), with increasingly diverse research paradigms addressing emerging topics such as hybrid training approaches, racial bias, and visual sampling strategies (Murray et al., 2024; Olma et al., 2024a, 2024b; Seitchik and Girgenti, 2025; Sudkamp et al., 2025) (see Table 2).

**Table 2:** Characteristics of included studies.

Author, year	Country	Study design	Groups	Sample size (n)	Gender (M/F)	Age (years)	Years of service (years)
Nieuwenhuys and Oudejans (2010)	Netherlands	Within-subject repeated measures	1. Low anxiety 2. High anxiety	7	6/1	23.8 ± 2.0	3.4 ± 2.4
Vickers and Lewinski (2012)	Canada	Quasi-experimental	1. Elite 2. Novice	Elite: 11 Novice: 13	Elite: all male Novice: 6/7	Elite: 38.82 ± 4.60 Novice: 30.54 ± 6.55	Elite: NR Novice: < 1.5
Nieuwenhuys et al. (2012)	Netherlands	Within-subject repeated measures	1. Low anxiety 2. High anxiety	36	33/3	37.79 ± NR	14.92 ± NR
Nieuwenhuys et al. (2015)	Netherlands	Randomized controlled trial	1. VBP-HT 2. VBP-LT 3. RLP-HT 4. Control	VBP-HT: 16 VBP-LT: 18 RLP-HT: 13 Control: 10	VBP-HT: 14/2 VBP-LT: 17/1 RLP-HT: 12/1 Control: 9/1	VBP-HT: 38.07 ± 8.66 VBP-LT: 37.41 ± 10.72 RLP-HT: 36.54 ± 11.38 Control: 37.20 ± 8.90	VBP-HT: 15.73 ± 9.76 VBP-LT: 14.29 ± 9.33 RLP-HT: 13.19 ± 12.24 Control: 12.90 ± 9.09
Landman et al. (2016)	Netherlands	Quasi-experimental	1. General police 2. Pre-selection 3. Arrest team	General: 18 Pre-selection: 24 Arrest team: 17	All male	General: 34.6 ± 9.44 Pre-selection: 29.4 ± 3.07 Arrest team: 30.6 ± 5.03	General: 13.22 ± 12.16 Pre-selection: 7.06 ± 3.63 Arrest team: 9.65 ± 4.39
Martaindale (2021)	USA	Randomized controlled trial	1. Training group 2. Control group	Control: 44 Training: 43	Control: 18/26 Training: 20/23	Control: 22.66 ± 5.83 Training: 22.05 ± 3.63	Police academy trainees
Heusler and Sutter (2022b)	Germany	Quasi-experimental	1. Intervention 2. Control	Intervention: 35 Control: 25	Intervention: 25/10 Control: 20/5	NR	Police academy trainees
Huhta et al. (2022)	Finland	Quasi-experimental	1. Early novice 2. Intermediate novice 3. Expert	Early: 10 Intermediate: 13 Expert: 11	Early: 4/6 Intermediate: 8/5 Expert: 11/0	Early: 25.6 ± 3.4 Intermediate: 24.6 ± 4.4 Expert: 41.6 ± 4.3	Early: < 1.5 Intermediate: 8.0 ± 2.2 Expert: 16.7 ± 3.9
Heusler and Sutter (2022a)	Germany	Quasi-experimental	1. TU 2. MP 3. UP	TU: 12 MP: 12 UP: 13	TU: 9/3 MP: 9/3 UP: 10/3	TU: 32.0 ± 4.88 MP: 32.0 ± 5.14 UP: 29.0 ± 7.04	TU: 8.0 ± 3.18 MP: 9.0 ± 5.43 UP: 5.0 ± 7.39
Tawa (2023)	USA	Quasi-experimental	1. High racial essentialism 2. Low racial essentialism	49	34/15	NR	Police academy trainees
Murray et al. (2024)	USA	Cluster analysis	1. Efficient scanning 2. Inefficient scanning	44	NR	32.86 ± 7.2	7.05 ± 6.16
Olma et al. (2024b)	Germany	Randomized controlled trial	1. Intervention 2. Control	Intervention: 22 Control: 23	31/14	Control: 34.7 ± 5.3 Intervention: 36.0 ± 4.7	Control: 12.8 ± 7.9 Intervention: 13.2 ± 6.6
Olma et al. (2024a)	Germany	Randomized controlled trial	1. Intervention 2. Control	Intervention: 27 Control: 25	31/21	Control: 23.4 ± 3.5 Intervention: 23.0 ± 3.0	Police academy trainees
Seitchik and Girgenti (2025)	USA	Correlational (single group)	—	106	21/85	20.23 ± 3.14	Police academy trainees
Sudkamp et al. (2025)	UK	Quasi-experimental	1. Expert 2. Novice	Expert: 10 Novice: 12	Expert: all male Novice: 10/2	Expert: 50.6 ± 3.5 Novice: 31.9 ± 3.4	Expert: 19.6 Novice: 0

VBP-HT = video-based simulation with high-threat training; VBP-LT = video-based simulation with low-threat training; RLP-HT = real-life practice with high-threat training; TU = tactical unit officers; MP = matched patrol officers; UP = unmatched patrol officers. Some studies did not report specific relevant data (indicated as “NR”).

#### 3.2.2 Geographic distribution of studies

In terms of geographic distribution, research institutions were primarily concentrated in the Netherlands (*n* = 4), Germany (*n* = 4), the United States (*n* = 4), the United Kingdom (*n* = 1), Finland (*n* = 1), and Canada (*n* = 1). Research teams in the Netherlands (Nieuwenhuys and Oudejans, 2010; Nieuwenhuys et al., 2012, 2015; Landman et al., 2016) have developed a systematic body of work examining the effects of stress and anxiety on shooting performance. German researchers (Heusler and Sutter, 2022a, 2022b; Olma et al., 2024a, 2024b) have made significant contributions to the study of tactical gaze control and training interventions. Studies from the United States (Vickers and Lewinski, 2012; Martaindale, 2021; Tawa, 2023; Murray et al., 2024; Seitchik and Girgenti, 2025) have led advancements in real-world eye-tracking applications and decision-making paradigms. Meanwhile, research from the United Kingdom (Sudkamp et al., 2025) has provided novel insights into visual sampling strategies among authorized firearms officers. Notably, there is a clear trend toward interdisciplinary collaboration, with contributions from psychology, sport science, criminal justice, human factors engineering, and policing practice. This cross-disciplinary integration has collectively driven the multidimensional development of the field (see Table 2).

#### 3.2.3 Sample characteristics

The 15 studies included in this review exhibit considerable diversity in sample characteristics, with sample sizes ranging from 7 to 122 participants. In terms of sample composition, active-duty police officers constitute the primary population, including general patrol officers, tactical unit officers, authorized firearms officers, and arrest team members. Police trainees represent the second-largest group, primarily drawn from police academies in Germany and Finland. University students, typically from criminal justice or psychology programs, were also included in some studies.

Regarding age distribution, the mean age of active-duty police samples ranged from approximately 20 to 50.6 years. Novice officers (including trainees and newly recruited personnel) tended to be younger (approximately 23–32 years), whereas experienced officers (with more than 12 years of service on average) were older (approximately 35–50 years). Tactical unit officers had a mean age of 32–41 years, which is notably higher than that of general patrol officers (29–32 years), reflecting the substantial experience typically required for selection into specialized units.

In terms of gender distribution, the overall male-to-female ratio across the 15 studies was approximately 4.7:1, broadly reflecting the gender composition of police forces. However, some groups (e.g., expert officer samples) included no female participants, indicating that gender representation remains limited in certain contexts.

With respect to years of service, the average policing experience among active-duty officers ranged from 3.4 to 19.6 years. General patrol officers typically had 3–13 years of experience, while tactical unit officers averaged 8–19.6 years, and arrest team members ranged from 8 to 16.7 years. Notably, the operational definitions of “novice” and “expert” varied across studies. Some studies defined novices as police trainees (with less than 1.5 years of experience) and experts as tactical unit officers (with more than 15 years of service on average). Other studies classified untrained patrol officers as novices (mean experience of 5–9 years) and tactically trained officers as experts (mean experience of 8–19.6 years) (see Table 2).

### 3.3 Results of methodological quality assessment

Among the four randomized controlled trials (RCTs), all studies reported appropriate randomization procedures, comparable baseline characteristics between groups, and adequate implementation of intervention protocols. However, two studies did not provide complete outcome data, and blinding procedures were not adequately reported in some studies. Overall, the RCTs demonstrated relatively good methodological quality, with appraisal outcomes primarily ranging from moderate to moderate-to-high quality.

Among the six non-randomized studies, most studies demonstrated satisfactory performance in terms of participant representativeness, appropriateness of measurement methods, and completeness of intervention implementation. However, incomplete outcome data reporting was a common limitation, and some studies did not sufficiently describe strategies for controlling potential confounding factors. One study exhibited limitations related to insufficient sample representativeness and inadequate reporting of confounder management. Overall, the methodological quality of non-randomized studies was predominantly moderate, with some studies reaching a moderate-to-high level of quality.

Among the five quantitative descriptive studies, the measurement approaches and statistical analyses employed were generally appropriate. However, some studies provided insufficient information regarding sampling strategies and non-response bias, and several studies had limitations related to sample representativeness. Overall, methodological quality varied across quantitative descriptive studies, with appraisal ratings ranging from moderate-to-low to moderate-to-high quality.

Overall, the included studies demonstrated acceptable methodological quality. Nevertheless, several limitations were identified, particularly regarding outcome data completeness, reporting of blinding procedures, sample representativeness, and control of potential confounding factors. Detailed appraisal results are presented in Supplementary Table S1.

### 3.4 Core eye-tracking metrics in police use-of-force decision-making

#### 3.4.1 Eye-tracking devices

Across the 15 included studies, a total of six types of eye-tracking systems were employed. For screen-based (desktop-mounted) systems, the SR Eyelink 1,000 Plus (sampling rate: 500–1,000 Hz) and Tobii T60 (60 Hz) were primarily used in static scene perception tasks (Huhta et al., 2022; Seitchik and Girgenti, 2025). Head-mounted mobile eye trackers were the most widely used. Tobii Glasses 2 (50 Hz) were applied in both real-world scenarios and video-based simulations (Murray et al., 2024; Sudkamp et al., 2025). The Pupil Labs series (including Pupil Core and Pupil Invisible/Neon, 200 Hz) were utilized in dynamic video tasks and live-fire shooting scenarios (Heusler and Sutter, 2022a, 2022b; Olma et al., 2024a, 2024b). Earlier studies employed the Applied Science Laboratories Mobile Eye system (30 Hz) for shooting behavior research (Nieuwenhuys and Oudejans, 2010; Vickers and Lewinski, 2012). Additionally, SMI eye-tracking glasses (30 Hz) were used in shooting tasks under stress conditions (Landman et al., 2016). Embedded eye-tracking systems integrated within virtual reality head-mounted displays (VR HMDs, 30 Hz) were applied in high-ecological-validity simulation training environments (Martaindale, 2021; Tawa, 2023). Overall, sampling rates ranged from 30 Hz to 200 Hz. Devices with sampling rates of 200 Hz or higher provide greater precision in capturing rapid saccadic movements and pupil dynamics.

#### 3.4.2 Eye-tracking metrics

Based on frequency of use and functional roles in the literature, the eye-tracking and related metrics identified in this review were categorized into five groups: fixation-related metrics, saccade-related metrics, visual state and tactical behavior metrics, physiological–eye-tracking integrated metrics, and indirect behavioral metrics (see Table 3).

**Table 3:** Characteristics of the study intervention.

Category	Eye-tracking metrics	Operationalization/analytical strategy	Representative studies
Fixation-related metrics	Number of fixations; Fixation duration	Fundamental indicators used to assess visual search efficiency.	Sudkamp et al. (2025), Murray et al. (2024), Huhta et al. (2022)
	Fixation order	Weighted scoring (first fixation = 100, decreasing sequentially) to reflect priority of visual attention across areas in early perceptual processing; used to assess situational awareness.	Huhta et al. (2022)
	Quiet eye	Duration of the final fixation (≥100 ms) on a task-relevant target (e.g., suspect’s weapon/phone or officer’s weapon) prior to action initiation; a key indicator distinguishing experts from novices.	Vickers and Lewinski (2012)
	Fixation location distribution	Proportion of fixations within Areas of Interest (AOIs), used to evaluate attentional allocation to threat-relevant regions (hands/waist) versus distractors (face).	Heusler and Sutter (2022b), Seitchik and Girgenti (2025)
	Fixation latency (time to first fixation)	Time from stimulus onset to first fixation on a target, reflecting efficiency of visual search.	Martaindale (2021)
Saccade-related metrics	Number of saccades; Mean saccade duration; Mean saccade amplitude; Peak saccadic acceleration	Jointly characterize the “deliberateness” versus “reactivity” of visual sampling. Efficient scanners exhibit fewer saccades, longer durations, larger amplitudes, and lower peak acceleration, indicating more planned rather than reactive search behavior.	Murray et al. (2024)
Visual state and tactical behavior metrics	Eye closure duration	Total time during which at least one eye is closed between threat onset and first shot, indicating interruptions in visual information acquisition.	Heusler and Sutter (2022a,b), Olma et al. (2024a)
	Muzzle position	Time at which the firearm is raised to eye level. Premature weapon elevation may occlude the officer’s view of the suspect’s hands/waist, delaying threat recognition.	Heusler and Sutter (2022a,b), Olma et al. (2024a)
	Head/body orientation	Assesses whether officers turn away or lower their head under stress (self-protective behavior), thereby restricting information intake.	Nieuwenhuys and Oudejans (2010)
Physiological–eye-tracking integrated metrics	Standard deviation of pupil diameter	Operationalized as an index of physiological stress; combined with Attentional Control Theory to explain how stress influences decision-making via attentional processes.	Tawa (2023)
	Heart rate	Collected synchronously with eye-tracking data to validate stress manipulation and examine the relationship between physiological arousal and gaze behavior.	Landman et al. (2016), Murray et al. (2024), Nieuwenhuys and Oudejans (2010), Nieuwenhuys et al. (2012)
Indirect behavioral metrics	Response time difference	Difference in reaction times between correct shootings (armed suspect) and erroneous shootings (unarmed suspect). Faster erroneous responses (~80 ms shorter) suggest decisions are based on expectancy rather than visual confirmation.	Nieuwenhuys et al. (2012), Nieuwenhuys et al. (2015)
	Shooting sensitivity	Derived from signal detection theory (along with shooting threshold), integrating hit and false alarm rates to assess threat detection ability and response bias.	Seitchik and Girgenti (2025), Tawa (2023)

### 3.5 Research paradigms in police use-of-force decision-making

#### 3.5.1 The “shoot/no-shoot” decision paradigm

The “shoot/no-shoot” paradigm simulates law enforcement scenarios in which participants are required to rapidly determine whether a suspect is armed and decide whether to shoot or withhold fire. When combined with eye-tracking technology, this paradigm enables the investigation of cognitive mechanisms underlying decision biases. Among the 15 studies included in this review, nine employed this paradigm to systematically examine gaze behavior and performance during decision-making (Nieuwenhuys et al., 2012, 2015; Vickers and Lewinski, 2012; Martaindale, 2021; Heusler and Sutter, 2022b; Tawa, 2023; Olma et al., 2024a, 2024b; Seitchik and Girgenti, 2025). Across these studies, video simulations, virtual reality environments, and live-fire scenarios were used to classify decision outcomes into four categories: correct shooting (shooting an armed suspect), correct non-shooting (withholding fire for an unarmed suspect), erroneous shooting (shooting an unarmed suspect), and erroneous non-shooting (failing to shoot an armed suspect). Eye-tracking metrics were integrated to reveal the cognitive mechanisms underlying these decision outcomes.

A consistent finding across studies is that accurate decision-making (both shooting and non-shooting) is closely associated with efficient and precise allocation of visual attention. Vickers and Lewinski (2012) reported that elite officers, when correctly shooting armed suspects, increased their fixation on the object held in the suspect’s hand (weapon/phone) from 18 to 71% across the final six fixations, reaching 86% in successful trials. Moreover, their quiet eye duration (318 ms) was significantly longer than that of novices (265 ms). Heusler and Sutter (2022b) further demonstrated that, following training interventions, police trainees showed substantial improvements in reaction time (from 969 ms to 551 ms) and time to first hit (from 1,143 ms to 654 ms) in correct shooting scenarios. In addition, the time required to raise the firearm to eye level was significantly reduced (from 2,250 ms to 247 ms), indicating a tighter coupling between visual processing and motor execution. In correct non-shooting decisions, Olma et al. (2024a, 2024b) reported that both experienced officers and trainees achieved near-perfect accuracy (100%) in non-threat scenarios. Their gaze behavior reflected cautious verification of harmless objects, suggesting effective visual confirmation processes.

In contrast, the cognitive mechanisms underlying erroneous decisions (both false-positive and false-negative responses) constitute a central focus of this research domain. Nieuwenhuys et al. (2012) found that under high-anxiety conditions, the rate of erroneous shootings increased from 11.81 to 18.29%. Notably, reaction times for erroneous shootings (approximately 380 ms) were significantly shorter than for correct shootings (approximately 461 ms), while eye-tracking measures (e.g., scan ratio, time to first fixation, fixation duration) showed no significant differences between high- and low-anxiety conditions. These findings suggest that erroneous shootings are driven not by insufficient visual information acquisition but by expectancy-driven processes, whereby officers initiate shooting responses before fully verifying visual information. Subsequent research by the same group (Nieuwenhuys et al., 2015) confirmed the robustness of this effect: even after 3 weeks of real-life training, the influence of threat expectancy on erroneous shooting did not diminish. From a gaze allocation perspective, Vickers and Lewinski (Vickers and Lewinski, 2012) further elucidated the mechanism of erroneous non-shooting. Novice officers exhibited late-stage gaze shifts toward their own weapon in 84% of trials, resulting in insufficient fixation on the suspect’s handheld object (only 34%). This attentional misallocation contributed to high error rates, particularly in phone scenarios, where erroneous shooting reached 61.5% among novices (compared to 18.2% among elite officers).

Another critical line of research concerns the moderating role of racial bias in decision-making. Seitchik and Girgenti (2025) demonstrated that racial stereotypes significantly influence both decision outcomes and gaze allocation. During practice trials, participants showed stereotype-consistent bias, with faster and more accurate rejection of unarmed White targets compared to unarmed Black targets. Eye-tracking results indicated greater fixation on the hands of armed Black targets and on the head region of armed White targets. However, during experimental trials, participants exhibited a counter-stereotypical pattern, with significantly higher rates of erroneous shooting toward unarmed White targets (*p* < 0.001). In these cases, longer fixation durations on both the hands and head of White targets suggest a potential overcorrection of implicit bias.

Tawa (2023) further revealed, in a VR-based study, that the interaction between racial essentialism beliefs and stress influences decision-making through attentional mechanisms. Specifically, individuals with stronger essentialist beliefs fixated longer on Black suspects under high-stress conditions, which was associated with increased erroneous shooting rates and reduced shooting sensitivity.

#### 3.5.2 The expert–novice paradigm in police use-of-force decision-making

The expert–novice comparison paradigm examines differences in gaze behavior between police officers with varying levels of experience, aiming to uncover disparities in visual search strategies, threat assessment abilities, and their underlying cognitive mechanisms. Among the 15 studies included in this review, six directly compared expert and novice officers in terms of decision-making patterns and eye movement characteristics (Vickers and Lewinski, 2012; Landman et al., 2016; Heusler and Sutter, 2022a; Huhta et al., 2022; Murray et al., 2024; Sudkamp et al., 2025), while two additional studies indirectly revealed experience-related differences through clustering analyses or grouping by experience level (Olma et al., 2024a, 2024b). These studies collectively investigated expert–novice differences across a continuum from static scene perception to dynamic, high-pressure decision-making contexts.

Fixation patterns and threat cue recognition. The most salient difference between expert and novice officers lies in the allocation of gaze toward tactically relevant areas. Heusler and Sutter (2022a) compared tactical unit officers (TU) with patrol officers (MP/UP) and found that TU officers spent significantly more time fixating on the hands/hip region (17,707 ms) than MP (12,476 ms) and UP (9,901 ms), while spending significantly less time on the face (2,632 ms vs. 8,638 ms and 7,914 ms, respectively). This difference was particularly pronounced in scenarios where suspects concealed their hands in pockets: TU officers’ fixation duration on the hands/hip region was 86% higher than MP and 104% higher than UP, whereas MP officers fixated on the face 6.8 times longer than TU officers. These findings suggest that patrol officers are more susceptible to bottom-up attentional capture by salient stimuli (e.g., faces), whereas tactical unit officers can exert top-down control to direct attention toward task-relevant regions. Similarly, Huhta et al. (2022), in a static scene perception task, found that expert officers spent significantly less time fixating on environmental regions (1.54 s) compared to early novices (1.99 s) and intermediate novices (2.05 s). Moreover, both expert and intermediate groups directed their gaze toward the hands earlier than early novices, indicating that experience facilitates earlier attentional prioritization of critical threat-related cues.

Fixation duration and quiet eye strategy. Vickers and Lewinski (2012) demonstrated that elite officers exhibit a “funneling” pattern of gaze in lethal force encounters: across the final six fixations, attention to the suspect’s handheld object (weapon/phone) increased from 18 to 71%, reaching 86% in successful trials. Their quiet eye duration (318 ms) was significantly longer than that of novices (265 ms). In contrast, novice officers displayed inefficient gaze behavior, with 84% of trials involving late-stage saccades toward their own weapon, resulting in insufficient fixation on the suspect’s handheld object (34%). Notably, in 50% of trials, novices fired without directly fixating on the suspect. Landman et al. (2016) further reported that arrest unit officers (AU) achieved significantly higher shooting accuracy under stress (81%) compared to regular officers (54%). Experts exhibited shorter fixation durations on the suspect’s firearm (328–312 ms vs. 459–504 ms), but longer aiming durations (207 ms vs. 137 ms), suggesting that they are less prone to attentional capture by threat stimuli and instead allocate more visual resources to goal-directed aiming processes.

Saccadic activity and visual search efficiency. Using cluster analysis, Murray et al. (2024) classified officers into “efficient scanning” and “inefficient scanning” groups. The efficient group demonstrated superior visual search characteristics, including fewer saccades (47.85 vs. 99.92), longer saccade duration (90.5 ms vs. 62.6 ms), larger saccade amplitude (18.0° vs. 5.55°), and lower peak acceleration (7,469°/s vs. 15,720°/s). This group also showed longer fixation durations on threat and potential threat areas, and shorter fixation on irrelevant cues (e.g., a truck). Importantly, 72% of the efficient group had tactical training experience (compared to 53% in the inefficient group), while years of service did not significantly differ between groups. This suggests that tactical training, rather than mere experience duration, is a stronger predictor of efficient gaze behavior under stress.

Situational awareness and anticipatory gaze. Sudkamp et al. (2025) examined gaze behavior in dynamic video scenarios, comparing expert authorized firearms officers (AFOs; mean experience = 19.6 years) with novices. When the suspect was temporarily occluded, experts maintained stable gaze patterns (consistent NSS), whereas novices showed a significant decline in gaze similarity to expert patterns (group × visibility interaction, *p* = 0.008). This finding highlights a core expert–novice distinction: experts rely on experience-based expectations to anticipate threat locations and maintain attention toward critical regions even in the absence of visual input, whereas novices depend more heavily on immediate visual information and show reduced gaze stability when information is disrupted. Additionally, during response phases requiring shooting decisions, experts significantly increased their fixation duration (*p* = 0.015), whereas novices showed a slight decrease, indicating that experts can deliberately prolong fixation under pressure to support fine-grained visual processing.

Moderating effects of experience and training implications. Olma et al. (2024a, 2024b) examined the moderating role of experience in training outcomes by comparing experienced officers (mean service = 13 years) with trainees. Experienced officers exhibited near-ceiling performance in decision accuracy (false-positive rate = 0), with training primarily improving execution speed (reaction time reduced from 969 ms to 551 ms). In contrast, trainees, despite lower baseline performance, showed substantial improvements in both reaction time and time to first hit following training. The authors suggest that the mechanisms underlying training benefits differ between groups: trainees consolidate strategies acquired during academy training, whereas experienced officers refine and recalibrate existing strategies. This finding aligns with Heusler and Sutter (2022b), who argued that training plays a more critical role than field experience in shaping efficient gaze control strategies. Although tactical unit officers typically have less routine patrol exposure, their intensive tactical training enables them to develop superior gaze patterns compared to patrol officers.

#### 3.5.3 The effects of stress on police use-of-force decision-making

The impact of stress on police decision-making represents a central issue in police psychology and cognitive ergonomics. Among the 15 studies included in this review, eight explicitly examined the effects of stress on use-of-force decisions and their underlying eye-tracking mechanisms (Nieuwenhuys and Oudejans, 2010; Nieuwenhuys et al., 2012, 2015; Landman et al., 2016; Heusler and Sutter, 2022a; Tawa, 2023; Murray et al., 2024; Sudkamp et al., 2025). These studies employed a range of stress-induction paradigms—including shootback cannons, live-fire conditions, physiological stress measurements, and high-fidelity simulated scenarios—to systematically investigate how stress influences decision behavior, gaze patterns, and cognitive processing.

Stress induction paradigms and manipulation checks. Existing research primarily utilizes three types of stress manipulation. Nieuwenhuys and Oudejans (2010), Nieuwenhuys et al. (2012, 2015) introduced a “shootback cannon” in video-based simulations, which fired plastic pellets at participants’ legs, inducing pain and effectively elevating anxiety levels (self-reported anxiety increased from 3.13 to 7.09, accompanied by significant increases in heart rate and mental workload). Landman et al. (2016) employed live-fire return conditions to induce stress, reporting a decline in shooting accuracy under high-pressure conditions (from 85.16 to 80.10%) alongside elevated heart rates. Murray et al. (2024), in a real high-threat scenario involving an armed attacker, observed a substantial increase in heart rate from approximately 86 bpm to 181 bpm (≈110% increase), confirming the ecological validity of the stress manipulation. Tawa (2023) utilized virtual reality scenarios and measured pupil diameter variability as an index of physiological stress, successfully eliciting measurable stress responses.

Negative effects of stress on decision-making. A consistent finding across studies is that stress significantly increases the likelihood of erroneous shooting decisions. Nieuwenhuys et al. (2012) reported that under high-anxiety conditions, the rate of erroneous shootings increased from 11.81 to 18.29%, nearly doubling. Although reaction times for correct shootings of armed suspects decreased by approximately 6% (from 493 ms to 467 ms), shooting accuracy declined by about 5% (from 83.71 to 78.14%). Follow-up research (Nieuwenhuys et al., 2015) confirmed the robustness of this effect: erroneous shooting rates increased from 9.74% (low threat) to 16.51% (high threat) in both pre- and post-tests, indicating that the influence of stress on decision-making is highly persistent. Landman et al. (2016) further demonstrated that the impact of stress on decision errors is moderated by experience level. While regular officers exhibited a significant increase in anxiety under high stress (from 3.07 to 4.65), arrest unit officers showed no significant change (1.88 to 1.99) and maintained stable shooting accuracy (81.6 to 81.4%). This suggests that experience can buffer the detrimental effects of stress on performance.

Effects of stress on gaze behavior and visual processing. The influence of stress on eye movements presents a complex pattern across studies. Nieuwenhuys and Oudejans (2010) found that under high-anxiety conditions, officers exhibited significantly increased blink frequency (from 1.03 to 3.10 blinks per trial), resulting in a rise in eye closure time from 0.65 to 2.63% of total task duration. Additionally, vertical head orientation decreased, and officers were more likely to turn their backs to the opponent while reloading. These findings indicate a reduction in visual information acquisition efficiency under stress. However, Nieuwenhuys et al. (2012) found no significant differences in key eye-tracking metrics—such as scan ratio, time to first fixation, and fixation duration—between high- and low-anxiety conditions during decision-making tasks. Based on this, the authors proposed that stress primarily influences decision-making through enhanced threat expectancy, rather than through changes in visual information acquisition. In other words, officers under stress may adopt a “shoot-first, verify-later” strategy. Tawa (2023) further demonstrated an interaction between stress and cognitive beliefs. Individuals with higher levels of racial essentialism exhibited longer fixation durations on Black suspects under high-stress conditions, which in turn increased erroneous shooting rates and reduced shooting sensitivity. In contrast, individuals with lower essentialist beliefs showed the opposite pattern, highlighting the moderating role of cognitive factors in stress effects.

Gaze stability under stress and expert advantage. Sudkamp et al. (2025) found that in high-pressure scenarios where the suspect was temporarily occluded, expert officers maintained stable gaze patterns (consistent with their internal gaze templates), whereas novices showed a significant decline in gaze similarity (group × visibility interaction, *p* = 0.008). This suggests that experts can sustain anticipatory gaze under stress, directing attention toward locations where threats are likely to emerge based on prior experience, while novices rely more heavily on immediate visual input and exhibit reduced stability when information is disrupted. Murray et al. (2024) further demonstrated that, although both efficient and inefficient scanning groups experienced similar levels of physiological stress (no significant differences in heart rate changes), the efficient group maintained focused attention on threat-relevant areas and exhibited more controlled saccadic patterns (fewer, longer, and larger saccades). Importantly, their return-fire rate (85.5%) was significantly higher than that of the inefficient group (50%). These findings suggest that the impact of stress on decision-making is not determined solely by physiological arousal, but rather by the individual’s ability to maintain efficient visual attention allocation under stress.

#### 3.5.4 Training interventions and their effects on police use-of-force decision-making

Training interventions based on eye-tracking technology represent a crucial approach to enhancing police decision-making in the use of force. Among the 15 studies included in this review, six explicitly examined the impact of training interventions on police use-of-force decisions and the underlying eye-movement mechanisms (Nieuwenhuys et al., 2015; Martaindale, 2021; Heusler and Sutter, 2022b; Murray et al., 2024; Olma et al., 2024a, 2024b). The first four studies employed active interventions with randomized controlled designs to evaluate the efficacy of different training types, whereas the latter two studies used comparative designs to indirectly reveal how training shapes eye-movement patterns and decision-making performance.

Short-term visual training based on deliberate practice. Martaindale (2021) developed a 20-min visual training program grounded in deliberate practice principles. Using a randomized controlled design, 87 participants were assigned to either a training group (identifying guns versus harmless objects) or a control group (identifying letters/vowels). Training difficulty progressed incrementally (static images → complex scene images → dynamic videos) with immediate feedback. Results indicated that, at post-test, the training group exhibited a significantly lower error rate (0.48) than the control group (1.34), with a large effect size (Cohen’s d = 0.95); fixation latency was faster in the training group (0.39 s vs. 0.46 s, d = 0.91), while decision speed did not differ significantly. Eye-tracking data revealed that the training group adopted a strategy of foveal fixation for accurate object identification, whereas the control group continued to rely on peripheral vision. This study was the first to demonstrate that a brief, 20-min deliberate visual training can significantly enhance weapon identification accuracy by improving foveal visual targeting efficiency.

Theory-driven training based on tactical gaze control and visual attention. Heusler and Sutter (2022b) implemented a 90-min intervention (30 min theory + 60 min practice) emphasizing situational awareness, tactical gaze control (fixating on hands/hips), and visual attention, while the control group received conventional shooting training focused on speed–accuracy trade-offs. Among police trainees, the intervention group showed a significantly higher decision improvement rate in shooting scenarios (34%) compared to the control group (12%). Reaction time decreased from 1,083 ms to 551 ms (improvement of 263 ms), and time to first shot improved from 1,143 ms to 654 ms (improvement of 489 ms), whereas no significant changes occurred in the control group. Moreover, the intervention group reduced the time to bring the weapon to eye level from 2,250 ms to 247 ms (reduction of 2,003 ms), with a trend toward shorter eye closure duration (190 ms → 113 ms). The authors attributed these effects to three levels: perceptual (shifting gaze from the face to hands/hips), tactical (maintaining a low muzzle until threat confirmation), and behavioral (keeping both eyes open under stress).

Blended training based on the Four-Component Instructional Design model (4C/ID). Olma et al. (2024a, 2024b) integrated intervention training within the 4C/ID framework to systematically examine effects on both experienced officers (mean service: 13 years) and trainees. Both samples demonstrated significant improvements in reaction time and time to first hit (experienced officers: 969 ms → 551 ms; 1,143 ms → 654 ms; trainees: 1,115 ms → 824 ms; 1,279 ms → 866 ms), while the control groups showed no improvement or deterioration. However, decision accuracy in both groups was near ceiling (false positive rate = 0), and no significant improvement was observed. The authors suggested that the underlying mechanisms differed by experience level: trainees consolidated strategies acquired during academy training, whereas experienced officers refined and updated existing strategies. This finding highlights the moderating effect of experience on training outcomes—while decision accuracy may stabilize with accumulated experience, execution efficiency can still improve, and targeted interventions are effective even for experts.

Limitations: skill specificity in decision-making and aiming. Nieuwenhuys et al. (2015) demonstrated the skill-specific nature of training effects. Fifty-seven officers were assigned to four groups (video + high threat, video + low threat, real-life scenario + high threat, control) and engaged in 3 weeks of realistic scenario practice. Results indicated that none of the training groups showed a significant reduction in erroneous shootings under high-pressure conditions compared to controls. The authors emphasized that decision-making training is less malleable than shooting accuracy training—decision-making involves threat anticipation and interpretive biases that are resistant to short-term practice, whereas visuomotor coordination skills can be significantly enhanced through stress-based training. This aligns with findings from Murray et al. (2024), who reported that high-efficiency scanning groups (72% with tactical training experience) differed significantly from low-efficiency groups (53% with tactical training) in saccade patterns, fixation allocation, and return-fire rates (85.5% vs. 50%), despite similar service duration. These results further underscore that tactical training experience, rather than mere time in service, is a stronger predictor of efficient eye-movement patterns under stress.

## 4 Discussion

This study provides a systematic review of 15 eye-tracking studies on police use-of-force decision-making, integrating findings across five dimensions: core eye-movement metrics, decision paradigms, expert–novice differences, stress effects, and training interventions. Overall, eye-tracking technology offers an objective tool for revealing how police officers perceive threats, make decisions, and execute actions under high-pressure conditions, yielding important insights for understanding the cognitive mechanisms underlying policing decisions and optimizing training programs.

The review demonstrates that eye-tracking research on police use-of-force decisions has evolved from simple measures such as fixation duration and count to a multi-level framework encompassing fixation allocation (hands/hips vs. face), saccade dynamics (frequency, duration, amplitude, peak velocity), visual state (eye closure duration, muzzle position), and physiological–eye movement coupling (pupil variability and heart rate). This evolution reflects an increasingly nuanced understanding that “how to look” and “where to look” are equally critical. Notably, weighted fixation sequences (Huhta et al., 2022) and recent fixation analyses (Seitchik and Girgenti, 2025) provide refined methods for capturing the ambiguity of natural visual search, while reaction-time differences and signal-detection metrics offer effective proxies for visual information reliance when eye-tracking data are unavailable (Vickers and Lewinski, 2012).

Regarding the cognitive mechanisms underlying “shoot/no-shoot” decisions, nine studies converge on the finding that efficient decision-making relies on precise foveal fixation on the suspect’s hand-held object during critical time windows, avoiding anticipatory “shoot first, look later” patterns and cue loss due to attentional shifts. Vickers and Lewinski (2012) identified elite officers’ “funnel-shaped” fixation focus and extended quiet eye, while Nieuwenhuys et al. (2012) reported the counterintuitive pattern that erroneous shots occurred 80 ms faster than correct shots. Together, these findings indicate that decision biases largely stem from insufficient visual information acquisition or interpretive errors. Martaindale (2021) brief visual training intervention further validated this mechanism: the training group reduced errors by two-thirds through faster foveal targeting, without affecting decision speed, suggesting that perception (“seeing”) and judgment (“deciding”) are separable and that training should prioritize improving visual localization efficiency.

Concerning expert–novice differences, six comparative studies revealed that expert officers’ key advantages include: earlier allocation of gaze to tactical regions such as hands/hips, reducing attention capture by salient but information-poor facial cues; maintenance of anticipatory fixations under stress and prolonged fixations prior to decision-making to support fine-grained visuomotor preparation; and efficient visual search achieved through fewer saccades, longer saccade durations, and larger amplitudes. Importantly, Murray et al. (2024) reported that 72% of officers in the high-efficiency scanning group had tactical training experience (versus 53% in the low-efficiency group), with no significant differences in service duration. Similarly, Heusler and Sutter (2022a) found that officers in tactical units, despite less field experience, exhibited superior fixation patterns compared to patrol officers after intensive training. These findings collectively indicate that targeted tactical training is more influential than accumulated service time in shaping efficient eye-movement patterns under stress, providing an evidence-based rationale for resource allocation in police training.

Regarding stress effects and plasticity, eight studies indicated that stress influences decision-making primarily via two pathways: (1) amplifying threat anticipation, prompting premature shooting responses before sufficient visual information is acquired (Nieuwenhuys et al., 2012); and (2) disrupting fixation stability, causing attention to shift from task-relevant regions to salient stimuli or irrelevant cues (Nieuwenhuys and Oudejans, 2010). However, the effects of stress on decision-making are highly skill-specific and experience-dependent. Nieuwenhuys et al. (2015) found that decision biases are remarkably stable and resistant to 3 weeks of realistic scenario practice, whereas shooting accuracy can be substantially improved through similar training. This suggests that aiming and decision-making should be treated as relatively independent skills, requiring distinct training strategies. Sudkamp et al. (2025) further demonstrated that expert officers can maintain anticipatory fixations under stress, whereas novices rely on immediate visual input, with fixation stability decreasing during information interruptions, indicating that stress resilience can be developed through training.

Regarding training effectiveness, six intervention studies validated three effective paradigms: short-term visual training based on deliberate practice (20 min), theory-driven training emphasizing tactical gaze control (90 min), and blended training based on the Four-Component Instructional Design (4C/ID) model. Common features across these interventions include the use of non-lethal weapons for safety, dynamic video scenarios to preserve ecological validity, and immediate feedback to facilitate strategy internalization. Nevertheless, training effects are highly skill-specific—once decision accuracy stabilizes with accumulated experience, interventions primarily improve execution efficiency rather than accuracy (Olma et al., 2024a, 2024b). These findings suggest that training goals should be tailored to experience level: novices should focus on decision accuracy (where and when to shoot), while experts should focus on execution efficiency (speed of shooting).

Furthermore, from a geographical perspective, current eye-tracking research on police use-of-force decision-making demonstrates a high degree of regional concentration. The 15 included studies were primarily conducted in the Netherlands, Germany, the United States, the United Kingdom, Finland, and Canada, with no studies identified from Asia, Africa, South America, or other non-Western countries. This geographical distribution highlights a potential structural gap in the current evidence base. Existing findings have been largely derived from policing systems and law enforcement cultures within Western democratic countries. However, legal frameworks governing the use of force, police training systems, firearm regulations, and patterns of police–citizen interactions may differ substantially across countries and regions. Consequently, the extent to which the visual attention strategies, stress-regulation mechanisms, and training intervention effects identified in this review can be generalized to different legal, institutional, and cultural contexts remains uncertain and requires further empirical validation. In particular, within diverse sociocultural environments, threat perception, use-of-force thresholds, and risk assessment strategies among police officers may be jointly shaped by institutional structures, cultural norms, and societal expectations. Future research should therefore expand participant recruitment across geographical regions, promote cross-cultural comparative investigations, and examine the relationship between eye-movement patterns and decision-making behaviors across different policing systems. Such efforts will be essential for improving the external validity, cross-cultural applicability, and international relevance of findings in this field.

This scoping review was not prospectively registered on PROSPERO or other relevant platforms. Although preregistration is not currently mandatory for scoping reviews, registering a review protocol can enhance methodological transparency and reproducibility. Due to the absence of a preregistered protocol, any modifications made during the review process could not be publicly documented, which may introduce potential reporting bias and represents a limitation of the present study. Second, several included studies recruited police academy cadets or criminal justice students as experimental participants (Martaindale, 2021; Tawa, 2023; Seitchik and Girgenti, 2025). Although these studies employed police use-of-force decision-making paradigms and examined visual attention and cognitive processing mechanisms relevant to law enforcement decision-making, these samples lacked substantial operational policing experience, which may limit the generalizability of findings to active-duty police officers. Future research should therefore include larger samples of serving officers and law enforcement trainees to enhance ecological validity. Finally, the included studies demonstrated some heterogeneity in age distribution. Several studies included participants with relatively broad age ranges (approximately 20–50 years), and advancing age may influence cognitive processes relevant to decision-making, including attentional resource allocation, visual search efficiency, and response speed. Therefore, the expert–novice differences or training effects identified in this review may be influenced by both occupational experience and age-related factors. As this study was designed as a scoping review, its primary purpose was to map the characteristics and development of the research field rather than to conduct quantitative effect estimation or causal inference regarding age-related effects. Consequently, the independent contribution of age to eye-tracking measures and use-of-force decision-making outcomes could not be determined. Future studies should employ age-matched expert–novice comparison designs or incorporate age as a covariate in statistical models to improve the interpretability and precision of findings.

## 5 Conclusion

This systematic review, based on 15 eye-tracking studies, comprehensively synthesizes research progress across five dimensions of police use-of-force cognitive decision-making: core eye-movement metrics, decision paradigms, expert–novice differences, stress effects, and training interventions. The core eye-movement framework has evolved from single fixation metrics to a multi-level integrated system encompassing fixation allocation, saccade dynamics, visual state, and physiological–eye movement coupling. Research on decision paradigms indicates that efficient decision-making hinges on precise foveal fixation on objects held by suspects, thereby avoiding anticipatory “shoot first, look later” behaviors. Expert–novice comparisons reveal that targeted tactical training shapes efficient eye-movement patterns under stress more effectively than mere cumulative experience. Stress influences decision-making by amplifying threat anticipation and disrupting fixation stability, yet these effects are skill-specific: decision biases are resistant to short-term training, whereas aiming efficiency can be substantially improved. Interventions based on deliberate practice, tactical gaze control, and instructional design frameworks have been shown to effectively enhance fixation efficiency and response speed. Future research should focus on developing unified training frameworks that integrate cognitive reappraisal and attentional control, translating laboratory findings into scalable police training practices. Such evidence-based interventions can reduce decision-making errors and enhance the safety of both officers and the public.

## Data Availability

The datasets presented in this study can be found in online repositories. The names of the repository/repositories and accession number(s) can be found in the article/Supplementary material.
