# Supplementary material for: Cognitive decision-making in police use of force: a scoping review based on eye-tracking research
Source: Front Psychol. 2026 Aug 12;17:1837475. doi: 10.3389/fpsyg.2026.1837475 (PMC13506682; doi:10.3389/fpsyg.2026.1837475)
Supplement: Supplementary file 1 [file Supplementary_file_1.DOCX]

| Database | Search strategy | amount |
| --- | --- | --- |
| Pubmed | | |
| #1 | Search: (((((eye tracking[Title/Abstract]) OR (eye movement[Title/Abstract])) OR (gaze tracking[Title/Abstract])) OR (gaze control[Title/Abstract])) OR (quiet eye[Title/Abstract])) OR (gaze analysis[Title/Abstract]) | 3452 |
| #2 | Search: ((((police[Title/Abstract]) OR (law enforcement[Title/Abstract])) OR (police officer[Title/Abstract])) OR (authorized firearms officer[Title/Abstract])) OR (active-duty police[Title/Abstract]) | 1818 |
| #3 | Search: (((((firearms use[Title/Abstract]) OR (weapon use[Title/Abstract])) OR (use of force[Title/Abstract])) OR (weapon deployment[Title/Abstract])) OR (shoot/don't shoot scenario[Title/Abstract])) OR (firearms operation[Title/Abstract]) | 4062 |
| #4 | #1 AND #2 AND #3 | 4 |
| EBSCO | | |
| #1 | SU (eye tracking) OR SU (eye movement) OR SU (gaze tracking) OR SU (gaze control) OR SU (quiet eye) OR SU (gaze analysis) | 81294 |
| #2 | SU (police) OR SU (law enforcement) OR SU (police officer) OR SU (authorized firearms officer) OR SU (active-duty police) | 191587 |
| #3 | SU (firearms use) OR SU (weapon use) OR SU (use of force) OR SU (weapon deployment) OR SU (shoot/don't shoot scenario) OR firearms operation | 1112 |
| #4 | (S3) AND (S2) AND (S1) | 0 |
| web of science | | |
| #1 | (((((TS=(eye tracking)) OR TS=(eye movement)) OR TS=(gaze tracking)) OR TS=(gaze control)) OR TS=(quiet eye)) OR TS=(gaze analysis) | 122584 |
| #2 | ((((TS=(police)) OR TS=(law enforcement)) OR TS=(police officer)) OR TS=(authorized firearms officer)) OR TS=(active-duty police) | 108744 |
| #3 | (((((TS=(firearms use)) OR TS=(weapon use)) OR TS=(use of force)) OR TS=(weapon deployment)) OR TS=(shoot/don't shoot scenario)) OR TS=(firearms operation) | 1039705 |
| #4 | #1 AND #2 AND #3 | 35 |
| Scoups | | |
| #1 | ( TITLE-ABS-KEY ( eye tracking ) OR TITLE-ABS-KEY ( eye movement ) OR TITLE-ABS-KEY ( gaze tracking ) OR TITLE-ABS-KEY ( gaze control ) OR TITLE-ABS-KEY ( quiet eye ) OR TITLE-ABS-KEY ( gaze analysis ) ) | 184111 |
| #2 | ( TITLE-ABS-KEY ( police ) OR TITLE-ABS-KEY ( law enforcement ) OR TITLE-ABS-KEY ( police officer ) OR TITLE-ABS-KEY ( authorized firearms officer ) OR TITLE-ABS-KEY ( active-duty police ) ) | 168823 |
| #3 | ( TITLE-ABS-KEY ( firearms use ) OR TITLE-ABS-KEY ( weapon use ) OR TITLE-ABS-KEY ( use of force ) OR TITLE-ABS-KEY ( weapon deployment ) OR TITLE-ABS-KEY ( shoot / don't shoot scenario ) OR TITLE-ABS-KEY ( firearms operation ) ) | 2298300 |
| #4 | #1 AND #2 AND #3 | 64 |
