# Supplementary material for: Cognitive decision-making in police use of force: a scoping review based on eye-tracking research
Source: Front Psychol. 2026 Aug 12;17:1837475. doi: 10.3389/fpsyg.2026.1837475 (PMC13506682; doi:10.3389/fpsyg.2026.1837475)
Supplement: Supplementary file 2 [file Table_1.DOCX]

### Supplementary Table S1 Quality assessment

●RCT Studies

| Study | Randomization | Baseline comparability | Complete outcome data | Blinding | Adherence | Summary of appraisal |
| --- | --- | --- | --- | --- | --- | --- |
| Arne Nieuwenhuys, 2015 | Yes | Yes | Yes | Not Report | Yes | Moderate–High |
| M. Hunter Martaindale, 2020 | Yes | Yes | Yes | Yes | Yes | Moderate–High |
| Joshua Olma, 2024 | Yes | Yes | No | Not Report | Yes | Moderate |
| Joshua Olma,2024b | Yes | Yes | No | Not Report | Yes | Moderate |

●Non-randomized Studies

| Study | Representativeness | Appropriate measurements | Complete outcome data | Confounders addressed | Intervention integrity | Overall appraisal |
| --- | --- | --- | --- | --- | --- | --- |
| Arne Nieuwenhuys, 2010 | Yes | Yes | No | Not Report | Yes | Moderate |
| Joan N. Vickers, 2012 | Yes | Yes | No | Not Report | Yes | Moderate |
| Arne Nieuwenhuys, 2012 | Yes | Yes | No | Not Report | Yes | Moderate |
| Annemarie Landman, 2015 | Yes | Yes | No | Yes | Yes | Moderate–High |
| Benedikt Heusler, 2022 | No | Yes | No | Not Report | Yes | Moderate–low |
| Benedikt Heusler, 2022b | Yes | Yes | No | Yes | Yes | Moderate–High |

●Quantitative Descriptive Studies

| Study | Sampling strategy | Representativeness | Appropriate measurements | Nonresponse bias | Statistical analysis | Overall appraisal |
| --- | --- | --- | --- | --- | --- | --- |
| Juha-Matti Huhta, 2022 | Not Report | Yes | Yes | Not Report | Yes | Moderate |
| John Tawa, 2023 | Not Report | No | Yes | Not Report | Yes | Moderate–low |
| Nicholas P. Murray, 2024 | Yes | Yes | Yes | Not Report | Yes | Moderate–High |
| Allison E. Seitchik, 2025 | Not Report | No | Yes | Not Report | Yes | Moderate - Low |
| Jennifer Sudkamp, 2025 | Yes | Yes | Yes | Not Report | Yes | Moderate–High |
